# Randomised controlled trial of mammographic screening in women from age 40: predicted mortality based on surrogate outcome measures

**DOI:** 10.1038/sj.bjc.6602395

**Published:** 2005-02-22

**Authors:** S Moss, M Waller, T J Anderson, H Cuckle, (writing committee) for the Trial Management Group

**Affiliations:** 1 Cancer Screening Evaluation Unit, Institute of Cancer Research, Brookes Lawley Building, 15 Cotswold Road, Sutton, Surrey SM2 5NG, UK; 2 Department of Pathology, Western General Hospital, Crewe Road, Edinburgh EH4 2XU, UK; 3 Department of Reproductive Epidemiology, University of Leeds, Leeds Screening Centre, 3 Gemini Park, Sheepscar Way, Leeds LS7 3JB, UK

**Keywords:** mammography, screening, breast, age, surrogate, mortality

## Abstract

A trial in the UK to study the effect on mortality from breast cancer of invitation for annual mammography from the age of 40–41, has randomised a total of 160 921 women in the ratio 1 : 2 to the intervention and control arms. All breast cancers diagnosed in the two arms have been identified, and the histology reviewed. This paper presents the results of an interim analysis using surrogate outcome measures to compare predicted breast cancer mortality in the two arms based on 1287 cases diagnosed to 31.12.1999. Due to earlier diagnosis, there is currently an 8% excess of invasive breast cancers in the intervention arm. The ratio of predicted deaths at 10 years in the intervention arm relative to the control arm, adjusted for this excess diagnosis, ranges from 0.89 (95% confidence interval (CI) 0.78–1.01) to 0.90 (95% CI 0.80–1.01). Screening from age 40 may result in a lower reduction in breast cancer mortality than that observed in other trials including women below age 50. This analysis based on surrogate outcome measures suggests that a reduction in breast cancer mortality may be observed in this trial. However, a number of assumptions have been necessary and firm conclusions must await the analysis of observed mortality from breast cancer.

At the time the NHS breast screening programme (NHSBSP) was introduced in 1988, evidence from the randomised controlled trials suggested that the benefit of screening was restricted to women aged 50 and over, and it was decided to include women aged 50–64 in the invitation system. The current Age Trial was established to investigate the benefit of screening in younger women, specifically the effectiveness of inviting women annually between the ages of 40 and 47–48.

The primary outcome measure of the trial is mortality from breast cancer. However, detailed pathology information on all breast cancer cases in the trial is also being collected, in order to permit an earlier analysis of surrogate outcome measures to be performed.

## MATERIALS AND METHODS

The methodology of the trial has been described in detail elsewhere (Moss, 1999). Briefly, 160 921 women have been randomised in the ratio 1 : 2 to an intervention arm and to a control arm. Randomisation is individual, stratified by GP practice. Women in the intervention arm are invited annually for screening by mammography (by two views at first screen, and one view thereafter unless otherwise indicated.) Women in the control arm receive usual medical care. The original protocol to offer each woman seven annual screens was subsequently extended to include invitations up to the calendar year of each woman's 48th birthday. Ethics approval was obtained from London (formerly North Thames) MREC.

### Sample size

The trial was designed to randomise 195 000 women aged 40–41 at entry, in order to have 80% power to detect a 20% reduction in breast cancer mortality at 10 years of follow-up in the intervention arm, at the 5% significance level, using a one-sided test. This was based on an estimated mortality of 3.3 per 1000 in the control arm in women free from breast cancer at entry into the trial (Moss, 1997). In 1999, recruitment was closed at 160 921 women due to difficulties in recruiting new centres, the last women having been randomised in 1997; the revised power is 73%.

### Trial population

**Figure 1:**
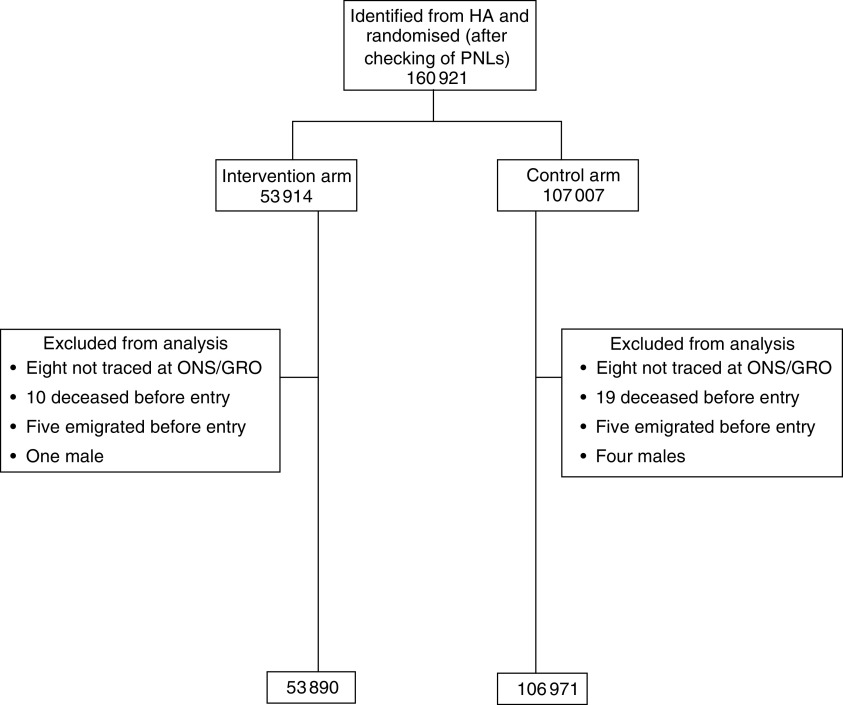
Flow diagram of the progress through the phases of the trial.

Recruitment of centres took place between 1991 and 1996 and includes 23 NHSBSP breast screening units in England, Wales and Scotland. Figure 1 shows the number of women randomised by trial arm; a total of 60 women have been excluded from analyses for reasons given in the flowchart. As at March 2002, women in the intervention arm had been offered a mean of 6.6 screens. Uptake of invitation is around 70%, and 84% of women randomised to the intervention arm were still invited at round 5. Details of uptake and screening outcomes are given elsewhere (Moss *et al*, 2005).

All women are ‘flagged’ at the NHS central register, which supplies information on all deaths and cancer registrations in the trial population. In addition, information on breast cancer cases is obtained from pathology laboratories and cancer registries and cross matched against the trial population database. Breast cancer cases included in this analysis are those diagnosed from trial entry up to 31.12.1999, a period for which ascertainment of cancers was estimated to be reasonably complete, based on age-specific incidence rates for England and Wales.

### Pathology data

For all trial cases identified, the original pathology report and representative histology slides are requested from the relevant laboratory. Each case is reviewed by a panel of three consultant histopathologists, and a consensus diagnosis reached on tumour size, nodal status, grade and histological type.

The review process has been described in detail elsewhere (Anderson *et al*, 2002). The pathology variables have been combined to calculate different prognostic indices. The Nottingham prognostic index (NPI) includes size, node status and grade according to the formula (0.2 × size (cm)+grade (1–3)+nodes (1–3), where 1=node negative, 2=1–3 nodes positive and 3⩾3 nodes positive) (Blamey, 1996). The index has previously been validated on series of clinical breast cancer cases using cutoff points for four prognostic groups (Blamey, 1996; Todd *et al*, 1997). Recently, the groupings have been reformulated for cases diagnosed since 1987 (post use of adjuvant therapy) and five prognostic groups identified; information on the 10-year survival of each group and on the baseline hazard and hazard ratios for individual components have been supplied by the Nottingham group (Sarah Pinder, personal communication).

Two other prognostic indices have also been proposed. The Swedish Two-County Studies Survival index uses information on size, lymph node status, grade and histological type, and has been developed using data from the randomised trial (Tabar *et al*, 1995b). The relative hazards of different factors, including lymph node status, have previously been validated against observed mortality reduction in the trial in different age-groups including women aged 40–49 (Tabar *et al*, 1995a). Updated values for the hazard ratios were obtained for this analysis (Duffy S, personal communication). Assuming a 10-year baseline death rate of 0.04, the hazard was calculated for each cancer, and 10-year survival estimates calculated for quantiles of this hazard in the total data set.

The EDCAT index was developed using data from the Edinburgh Randomised Trial of breast cancer screening, and includes size, histological type, histological grade and node status/number positive (Anderson *et al*, 2000). Size is included as six categories as opposed to a continuous variable (1–9, 10–14, 15–19, 20–29, 30–49 and 50+mm). Histological type is classified as 1=special, 2=non-special. Grade and nodal status are categorised as for the NPI. An index was formulated based on the hazard ratio from a multivariate analysis including all four factors, and survival associated with four groups (of equal numbers) calculated. A simplified index formula is 0.7 × size group+1 × type+1 × grade+1 × node group. Information on the baseline hazard and hazard ratios for individual components have been supplied by the investigators (Alexander F, personal communication).

For all the pathology variables in the present study, the proportion unknown varies between the control and intervention arm. However, for nearly all the invasive cases at least one of the variables was known: 1124 using NPI and 1126 using the Edinburgh index. For cases with missing data where at least one variable was known, we predicted the prognostic index for the NPI and Edinburgh index by fitting a regression model using those cases where information was available on all factors. Where all variables are unknown, the mean hazard of cases where at least one factor is missing has been used. The Swedish Two Counties model included a separate hazard ratio for cases where one or more components were missing.

### Statistics

Person-years were calculated from date of entry to date of death, date of diagnosis of *in situ* or invasive cancer, or to 31.12.1999, whichever was earliest.

For each of the prognostic indices, the predicted number of cancers in each arm surviving at 10 years was calculated by multiplying the 10-year survival rates by the numbers of cancers in each prognostic group and summing over all groups. Cancers with an unknown prognostic category were excluded from these calculations.

To take account of the excess of cancers in the intervention arm due to earlier diagnosis, the probability (*p*) of each screen-detected cancer remaining asymptomatic to the end of the follow-up period (31.12.1999 or date of death) was calculated using the formula *p*(*t*>*n*)=*e*^−*λn*^ where 1/*λ* is the mean sojourn time (MST), assuming the sojourn time to be exponentially distributed (Paci *et al*, 2004). This probability was summed for the screen-detected cancers in each prognostic category, and the total subtracted from the observed number of cancers. A mean sojourn time of 1.0 years for invasive cancers resulted in approximately equal incidence rates in the trial arms; sensitivity analyses were conducted using values of 0.75 and 1.25. When *in situ* cancers were included (for the analysis using the Swedish Two Counties index), a mean sojourn time of 1.75 years resulted in approximately equal rates; the sensitivity analysis used values of 1.5 and 2.0.

To take account of the effect of lead-time due to advancing the date of diagnosis by screening, the probability of death within 10 years of date of entry/randomisation has been calculated using the hazard ratios for the individual components for each of the three indices. These calculations used Cox proportional hazard models. In the case of the NPI and the Edinburgh index, these were applied to baseline hazard models. For the Swedish Two Counties model, the 10-year baseline death rate of 0.04 was taken and assumed to increase linearly with time. This yielded baseline survival estimates for each year of follow-up. Adjustment for excess diagnosis was made in this analysis by multiplying the probability of death for each screen-detected cancer by the probability of that cancer becoming symptomatic before the end of the follow-up period.

Variance for the relative risk was calculated using the formula given by Day and Duffy (1996) for each prognostic index (adjusted for the difference in the number of subjects in each arm).

## RESULTS

Of the 1303 cases identified in the trial population up to 31.12.1999, 1217 have been reviewed by the pathology review panel. For a further 31 the original pathology report was available, while for 55 it has not been possible to trace the original pathology report or hospital. A total of 16 cases included in analyses of breast cancer incidence (Moss *et al*, 2005) have been excluded here because the pathology review indicated that these were phyllodes tumour (4), sarcoma (1) or malignant lymphoma (1), or downgraded the lesion to ‘atypical hyperplasia’ (5) or benign (5). This leaves 1287 cases for inclusion in the analysis.

Table 1 shows numbers of breast cancers in each arm of the trial by invasive status and size category, by nodal status, grade and histological type for invasive cancers only. A total of 68 cases (50 in the control arm and 18 in the intervention arm) have been classified as ‘advanced’ by one of the review panels on the basis of information from the pathology report on clinical details (e.g. treatment by chemotherapy of large tumour, cytology/core biopsy only). These are included in the ⩾50 mm category in Table 1. For 47 cases no information on size was available.

The percentage of cases in each of the categories *in situ*/<10 mm, node negative and grade 1 is higher in the intervention arm than in the control arm. All these differences are highly significant (*P*<0.001). For all three factors there is a higher percentage of cases with missing data in the control arm.

Rates per 1000 women-years are given in Table 2 for all cancers and for invasive cancers only. Overall there is an excess of invasive breast cancers in the intervention arm (RR=1.08, 95% confidence interval (CI) 0.95–1.21). Including *in situ* cancers, the excess is 17% (RR 1.17, 95% CI 1.05–1.32).

Rates of cases ⩾20 mm are 12% lower in the intervention arm than in the control arm (RR 0.88, 95% CI 0.74–1.05). Rates of node-positive cancers are 11% lower (RR 0.89, 95% CI 0.72–1.10), but rates of grade 3 tumours are 6% higher (RR 1.06, 95% CI 0.88, 1.27) in the intervention arm. However, none of these differences is statistically significant.

Table 3 gives the numbers falling into the different categories of the three prognostic indices, and the predicted numbers surviving at 10 years from date of diagnosis, both for observed cases, and with the intervention arm adjusted for excess diagnosis. (The Swedish two Counties index includes *in situ* cases, whereas the other two indices are restricted to invasive cancers). Again, for each of the three indices there is a higher percentage of cases in the intervention arm in the best prognostic groups, and the proportion of cancers surviving at 10 years is higher in the intervention arm than the control arm. The percentage predicted to be surviving at 10 years in the intervention arm is lower when the numbers are adjusted for excess diagnosis due to the exclusion of a higher proportion of cases in the categories with better prognosis.

Table 4 gives the predicted deaths within 10 years of date of randomisation with the intervention arm adjusted for excess diagnosis in the intervention arm, together with the risk ratios for the intervention arm relative to the control arm.

There is a reduction in predicted breast cancer mortality in the intervention arm relative to the control arm of between 10 and 11% depending on the prognostic index used; the estimated reductions are of borderline statistical significance. The sensitivity analyses give ranges for the relative risk (95% CIs) of 0.89(0.79–1.00) –0.91(0.81–1.02) using the NPI, 0.88 (0.79–0.99)–0.90 (0.81–1.01) using the Edinburgh index and 0.88 (0.77–1.00)–0.90 (0.79–1.02) using the Swedish Two Counties index.

## DISCUSSION

The primary outcome measure of this trial is mortality from breast cancer in the two arms. This analysis of surrogate outcome measures predicts a reduction of 10–11% in breast cancer mortality in the intervention arm of the trial at 10 years from randomisation. The results are consistent for the three prognostic indices that have been used; however, there remains some uncertainty around these predictions.

Estimates of mortality reduction from screening in women under 50 have so far come primarily from subgroup analyses of randomised controlled trials recruiting cohorts of women from ages 40 or 45 upwards; meta-analyses of these trials have estimated a reduction of 18% at an average of 12.7 years of follow-up in women aged 40–49 at entry (Hendrick *et al*, 1997), but there remains debate as to how much of this effect is due to screening occurring after age 50 (de Koning *et al*, 1995). The results of these trials are summarised in Table 5, to allow comparison with the UK trial.

There are a number of possible reasons why the effect of screening on mortality in the current trial may be less than that expected on the basis of other randomised trials. The present trial is unique in inviting a cohort of women annually from age 40, and none of the cancers in the present analysis was diagnosed over the age of 50. Sensitivity of screening is likely to be lower at younger ages, due to increased breast density; our estimate of sensitivity of the first screening round is 74%, and 47–64% at later screens (Moss *et al*, 2005); sensitivity in the Swedish Two County study in women aged 40–49 has been estimated as 72–83% (Duffy *et al*, 1996) and in the Gothenburg trial as 84% (Bjurstam *et al*, 1997). Uptake at first screen was 70% in our trial, compared to an uptake of over 80% in the Swedish trials. These factors are likely to reduce the impact of screening on mortality.

The attraction of evaluating screening trials using surrogate end points lies in the ability to conduct earlier analyses, and in more precise estimates of mortality reduction, although the latter assumes no error in the survival probabilities. However, there may be limitations to predicting mortality in this trial using the NPI, as it was developed using a clinical series and largely in older age groups. Nevertheless, the coefficients used here for the NPI are those based on post-1987 cases (after the introduction of adjuvant therapy), and this may explain the greater similarity in outcome prediction between NPI and the Edinburgh index observed here than when both were applied to the Edinburgh randomised trial (Anderson *et al*, 2000). A number of other potential problems with the use of surrogate end points have been discussed by Morrison (1991), such as variation of prognostic factors within measurement categories, most of which would tend to lead to an underestimate of the effect of screening. The analysis of the Swedish Two County Study found that the predicted effect on mortality in the age group 40–49 years was conservative compared with that observed (Tabar *et al*, 1995b). The authors hypothesised that this might be attributable to an observed excess of cancers in the intervention arm, possibly due to over diagnosis in this age group, or the existence of a subgroup of women with a long sojourn time. Thus, the mortality reductions predicted here may underestimate the true reduction.

Ideally, predictions based on surrogate outcomes should be based on equivalent numbers of cases in the two arms. In the current analysis, there is an 8% excess of invasive breast cancers in the intervention arm (17% if *in situ* cancers are included). An excess is likely to remain until women in the control arm are invited for screening as part of the national programme at ages 50–52. The adjustment to predict mortality at 10 years from the date of randomisation as opposed to 10 years from the date of diagnosis will take account of lead-time bias due to the advancement in date of diagnosis of screen-detected cancers. We have attempted to adjust for excess diagnosis, and the effect of this adjustment does not vary greatly with different assumptions concerning the mean sojourn time. Analyses without this adjustment result in lower estimates of mortality reduction ranging between 4 and 6% (data not shown).

There is a 12% reduction in the rate of cancers ⩾20 mm in the intervention arm (equivalent to an 18% reduction adjusted for excess diagnosis), and a 11% reduction in node-positive rate (equivalent to a 17% reduction adjusted for excess diagnosis); both these measures have been shown to have a direct relationship with actual relative mortality reduction in randomised trials (Tabar *et al*, 1996).

In conclusion, results so far suggest that a reduction in breast cancer mortality in the trial is likely to be observed; however, the size of the reduction is uncertain and awaits definite results on mortality. The first such analysis will be performed when data are available on a mean follow-up of 10 years. Comparison of observed and predicted mortality reductions in this trial (and in the frequency trial (The Breast Screening Frequency Trial Group, 2002)) may provide further insight into the application of surrogate outcome measures. When all women in the control arm have been invited for their first screen at ages 50–52, we should be in a position to make a more accurate prediction of the long-term effect on mortality.

## Figures and Tables

**Table 1:** Tumour size, node status and grade by trial arm

	**Frequency (%)**	
	**Intervention arm**	**Control arm**
*Size (all cancers)*		
All CIS	69 (14.7)	54 (7.0)
1–9 mm	52 (11.1)	59 (7.6)
10–14 mm	81 (17.3)	124 (16.1)
15–19 mm	95 (20.3)	149 (19.3)
20–29 mm	98 (20.9)	209 (27.1)
30–49 mm	40 (8.5)	100 (13.0)
⩾50 mm	33 (7.1)	77 (10.0)
Unknown	10	37
Total	478	809
		
*Node status (invasive cancers)*		
Negative	210 (54.5)	306 (45.7)
1–3 positive	87 (22.6)	181 (27.0)
⩾4 positive	37 (9.6)	95 (14.2)
Not sampled	51 (13.2)	88 (13.1)
Not known	24	85
Total	409	755
		
*Grade (invasive cancers)*		
I	53 (13.6)	53 (7.6)
II	151 (38.8)	285 (40.9)
III	172 (44.2)	324 (46.5)
Not assessable	13 (3.3)	36 (5.2)
Unknown	20	57
Total	409	755
		
*Histological type (invasive cancers)*		
Special	46 (11.9)	74 (10.5)
Part special	38 (9.8)	62 (8.8)
Not special	300 (77.7)	556 (79.1)
Rare^a^	2 (0.5)	11 (1.6)
Unknown	23	52
Total	409	755

Advanced, Post Chemotherapy, Core biopsy, Diagnosis at death and Cytology only cancers were put in the 50+ mm category. Specimens larger than the slide were put in the 30–49 mm category.

a Rare includes invasive micropapillary, metaplastic carcinoma, atypical medullary and spindle cell tumour.

**Table 2:** Rates of invasive and all breast cancers by study arm

	**Intervention arm (312 957 w. years^a^)**		**Control arm (622 127 w.years^a^)**	
	***n***	**Rate per 1000 w.years**	***n***	**Rate per 1000 w.years**
All cancers	478	1.53	809	1.30
Invasive cancers	409	1.31	755	1.21
Invasive cancers ⩾20 mm	171	0.55	386	0.62
Node positive	124	0.40	276	0.44
Grade III	172	0.55	324	0.52

a To 31.12.1999, censored at date of death or diagnosis of breast cancer.

**Table 3:** NPI, Edinburgh and S2C categories and predicted 10-year survival from date of diagnosis by trial arm

		**Intervention arm**				**Control arm**	
				**(Adjusted for excess diagnosis)^a^**			
**Prognostic index**	**Predicted 10-year survival (%)**	**Cancers**	**Predicted number surviving at 10 years (%)**	**Cancers**	**Predicted number surviving at 10 years (%)**	**Cancers**	**Predicted number surviving at 10 years (%)**
*NPI*							
0–2.4(excellent)	98	31	30.4	27.1	26.6	28	27.4
2.4–3.4 (good)	90	84	75.6	71.5	64.4	101	90.9
3.4–4.4 (moderate I)	83	118	97.9	107.2	89.0	211	175.1
4.4–5.4 (moderate II)	75	88	66	83.6	62.7	211	158.3
⩾5.4 (poor)	47	79	37.1	75.2	35.4	173	81.3
Not known		9		9		31	
Total		409	307 (76.8)	373.7	278.1 (76.3)	755	533 (73.6)
							
*Edinburgh index*							
0–6.1	96.6	71	68.6	59.8	57.8	77	74.4
6.1–7.5	90.91	96	87.3	84.8	77.0	153	139.1
7.5–8.8	80.21	83	66.6	76.9	61.6	164	131.5
⩾8.8	60.77	150	91.2	143.3	87.1	332	201.8
Not known		9		9		29	
Total		409	313.7 (78.4)	373.7	283.5 (77.7)	755	546.8 (75.3)
							
*S2C Group*							
0–3.30	93.7	149	139.6	112.0	104.9	138	129.3
3.30–6.00	83.9	85	71.3	67.5	56.6	143	120.0
6.00–12.47	74.2	142	105.4	125.1	92.8	292	216.7
⩾12.47	37.3	93	34.7	88.5	33.0	207	77.2
Not known		9		9		29	
Total		478	351.0 (74.8)	402.2	287.3 (73.1)	809	543.2 (69.6)

a Assuming MST of 1.0 years for NPI, and EPI and MST of 1.75 for S2C.

**Table 4:** Predicted deaths in 10-year period from date of entry to trial^a^

	**Predicted deaths**		
	**Intervention arm *N*=53 890**	**Control Arm *N*=106 971**	**RR (95% CI)**
Nottingham prognostic index	72.6	159.9	0.90 (0.80, 1.01)
Edinburgh prognostic index	76.8	170.8	0.89 (0.80, 1.00)
Swedish two Counties index	74.0	165.2	0.89 (0.78, 1.01)

a Adjusted for excess diagnosis, using MST of 1.0 for NPI and EPI, 1.75 for S2C.

**Table 5:** Randomised controlled trials: women aged 40–49 at entry

	**Screening interval (months)**	**RR (95% CI) of breast cancer mortality in intervention arm**	**Length of follow-up**	**Uptake (Rd 1) (%)**
HIP study(20)	12	0.77 (0.53, 1.11)	18	67
Edinburgh(21)^a^	24	0.83 (0.54, 1.27)	14	61
Kopparberg(22)	24	0.76 (0.42, 1.40)	17	89
Ostergotland(23)	24	1.05 (0.64, 1.71)	17	89
Malmo I(23)^a^	18–24	0.74 (0.44, 1.25)	19	74
Malmo II(23)^b^	18–24	0.64 (0.39, 1.06)	9	75–80
Stockholm(23)	28	1.52 (0.89, 2.88)	15	82
Gothenburg(23)	18	0.58 (0.35, 0.96)	13	84
CNBBS1(24)	12	0.97 (0.74, 1.27)	11	100^c^

a Women aged 45–49 at entry.

b Women aged 43–49 at entry.

c Preselection by attendance for CBE.
